# Robotics and machine learning approaches to improve robustness of USgFUS: FUTURA

**DOI:** 10.1186/2050-5736-2-S1-A25

**Published:** 2014-12-10

**Authors:** Arianna Menciassi, Andreas Melzer, Erik Dumont, Matteo Santoro

**Affiliations:** 1 The BioRobotics Institute, Scuola Superiore Sant’Anna, Pisa, 56025, Italy; 2 University of Dundee, Dundee, DD1 4HN, UK; 3 Image Guided Therapy, Pessac, 33600, France; 4 Camelot Biomedical Systems, Genoa, 16152, Italy

## Background

Surgical robotics, machine learning for medical imaging, and focused ultrasound surgery (FUS) have the same long term goals: reducing invasiveness of therapies, augmenting the number of treatable diseases and, more in general, helping the healthcare system in terms of costs and benefits. Potentials from synergies among these fields are undeniable; cross-fertilization are expected to advance the applicability, robustness and safety of non-invasive therapies.

Ultrasound guided FUS is a promising strategy for the treatment of pathologies in the abdomen. However, some limitations dramatically slow a widespread diffusion in operating rooms, such as the limited accuracy, the 2D monitoring and the time cost for registration and repositioning.

## Materials and Methods

**Figure 1:**
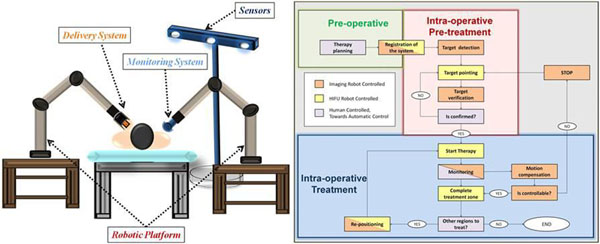
Platform and workflow proposed.

We created a collaborating network among EU recognized specialists on these fields, which turned into a Consortium for a project in the FP7, Theme 3: “Information and Communication Technologies”, Challenge 2: “Cognitive Systems and Robotics”. The proposed platform of the FUTURA project and workflow are shown in Figure 1.

Two robotic arms, one holding a 3D US-imaging probe, the other a FUS transducer with an integrated 2D imaging system, co-operate to point the target and monitor the therapy. Cognitive processes will be embedded in all the steps of the procedure, from planning to targeting, monitoring, control and verification of the therapy. Main figures of merit of the proposed platform are expected to be:

• Improved flexibility thanks to separation of imaging probe from therapeutic transducer and the possibility to position them accurately on the patient (no environmental constraints);

• 3D US therapy monitoring and motion compensation;

• Simultaneous repositioning, feedback control and therapy in order to considerably shorten treatment duration.

## Results

The project, whose acronym is FUTURA (Focused Ultrasound Therapy Using Robotic Approaches) started on Nov. 1, 2013 and will last 3 years. The website is the following: http://www.futuraproject.eu/.

## Conclusions

FUTURA is expected to advance collaboration among the above mentioned fields. The success of the proposal, submitted to a purely robotic call, shows an interest from that robotic community to the topic of non-invasive FUS. The results, which are expected to be disseminated to a very large audience (in terms of competences and scientific interests), will possibly widespread the concept of FUS as a real disruptive innovation for healthcare.

